# Supplementary material for: Functional Spectrum of USP7 Pathogenic Variants in Hao-Fountain Syndrome: Insights into the Enzyme’s Activity, Stability, and Allosteric Modulation
Source: bioRxiv. 2025 Mar 20:2025.03.20.644318. Preprint. [Version 1] doi: 10.1101/2025.03.20.644318 (PMC11957113; doi:10.1101/2025.03.20.644318)
Supplement: Supplement 1 — Table S1. Summary of enzyme kinetics and ubiquitin-binding affinities of USP7 mutations associated with Hao-Fountain syndrome. Figure S1. NMR spectra of USP7 variants associated with Hao-Fountain syndrome. 2D 15N TROSY spectra of 15N-labeled USP7 catalytic domain and its Hao-Fountain syndrome variants. Individual spectra are labeled with the corresponding variant name. Figure S2. Effect of Hao-Fountain syndrome variants on USP7 activity. Michaelis-Menten plots of the initial velocity (V0) as a function of ubiquitin-AMC concentration shown for (A) USP7 catalytic domain and its mutants and (B) FL-USP7 and its mutants. Figure S3. Ubiquitin binding to catalytic domains of USP7. Top: 15N TROSY spectra of the 15N-labeled USP7 catalytic domain and its mutants gradually titrated with unlabeled ubiquitin. Residue A381 is showcased for each spectrum. USP7:ubiquitin molar ratios are shown. Bottom: Plot showing the global chemical shift perturbations (Δω) in the spectra as a function of ubiquitin concentration, used to estimate the binding affinities for each USP7 variant (KD). Figure S4. MS-8 enhances the activity of USP7 variants. Comparison of the time course of deubiquitination reaction for FL-USP7 variants alone (red) and treated with 250 μM MS-8 (blue). The untreated WT curve is shown for reference (black). 0.1 nM USP7 was used with 500 nM ubiquitin-rhodamine as its fluorogenic substrate. [file media-1.pdf]

Figure S1

A

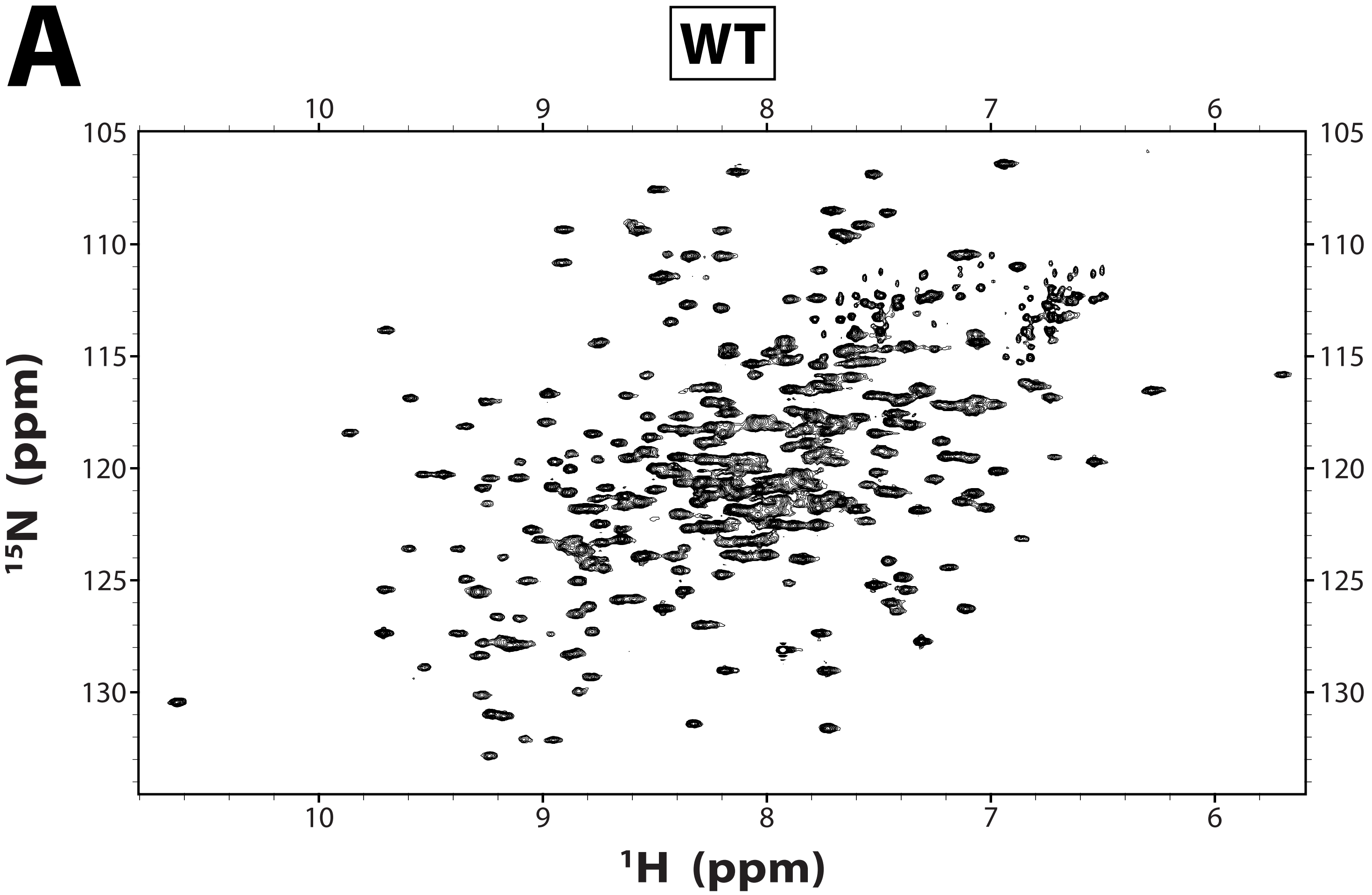

Figure S1

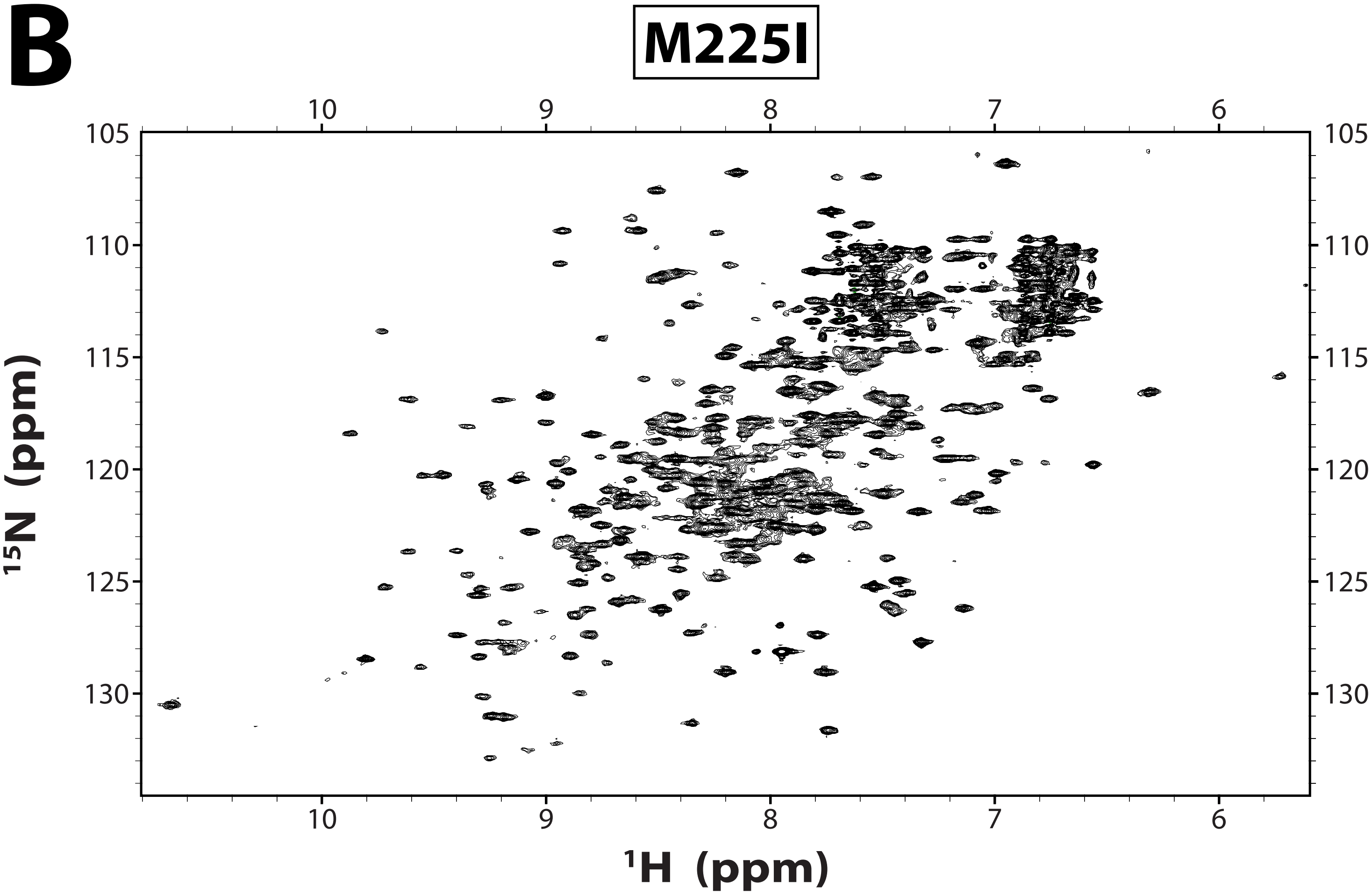

Figure S1

C

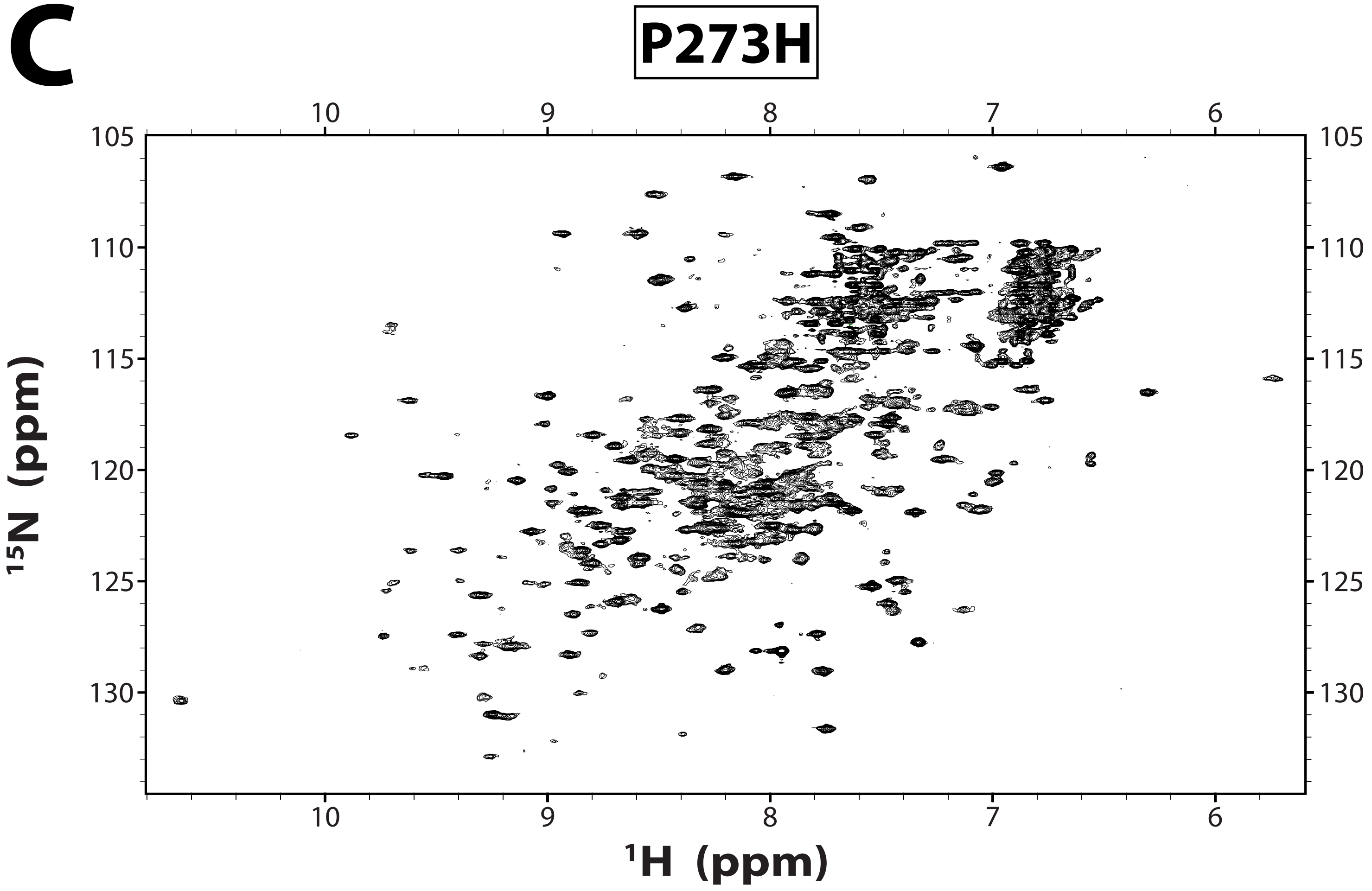

Figure S1

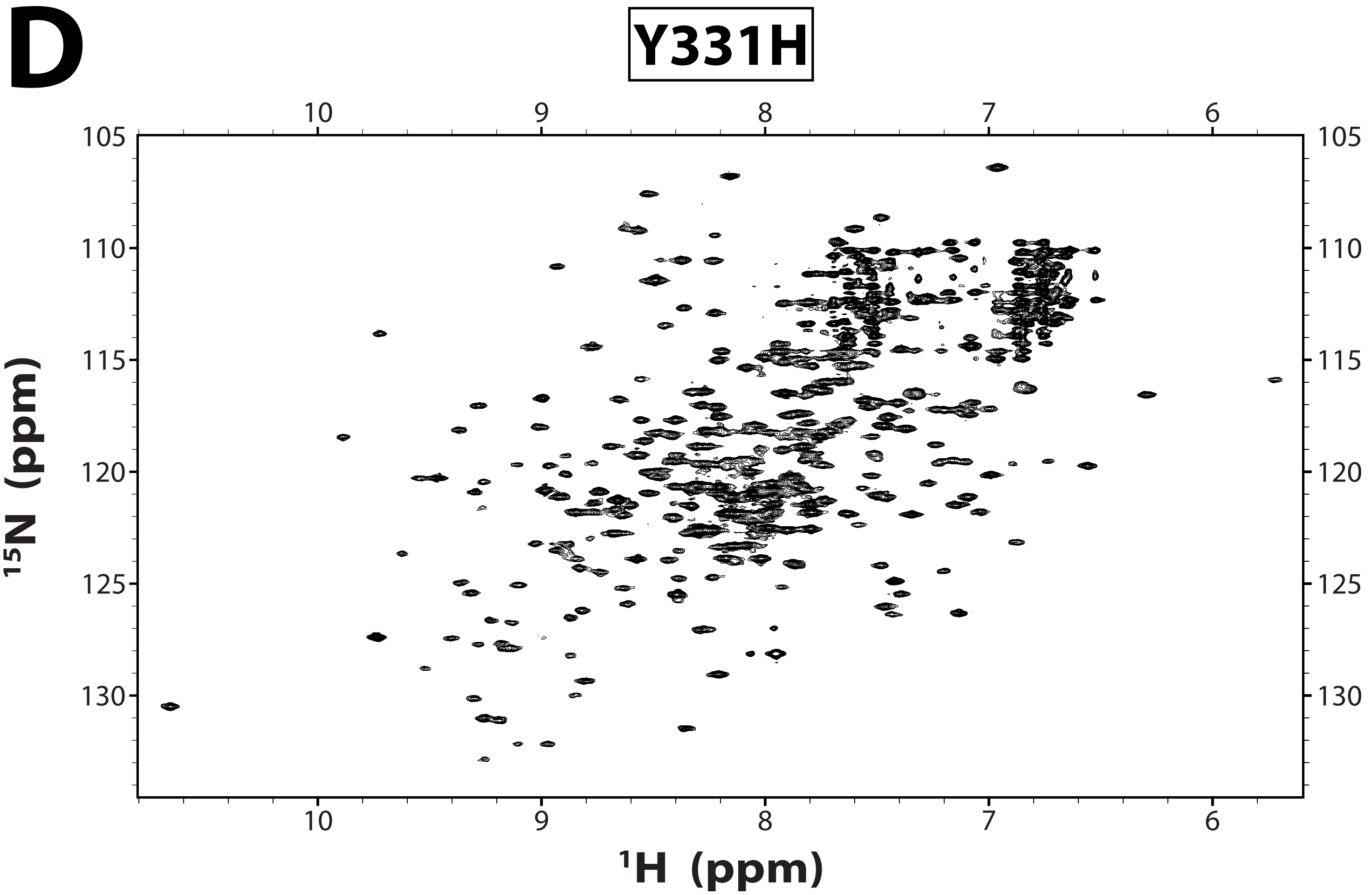

Figure S1

E

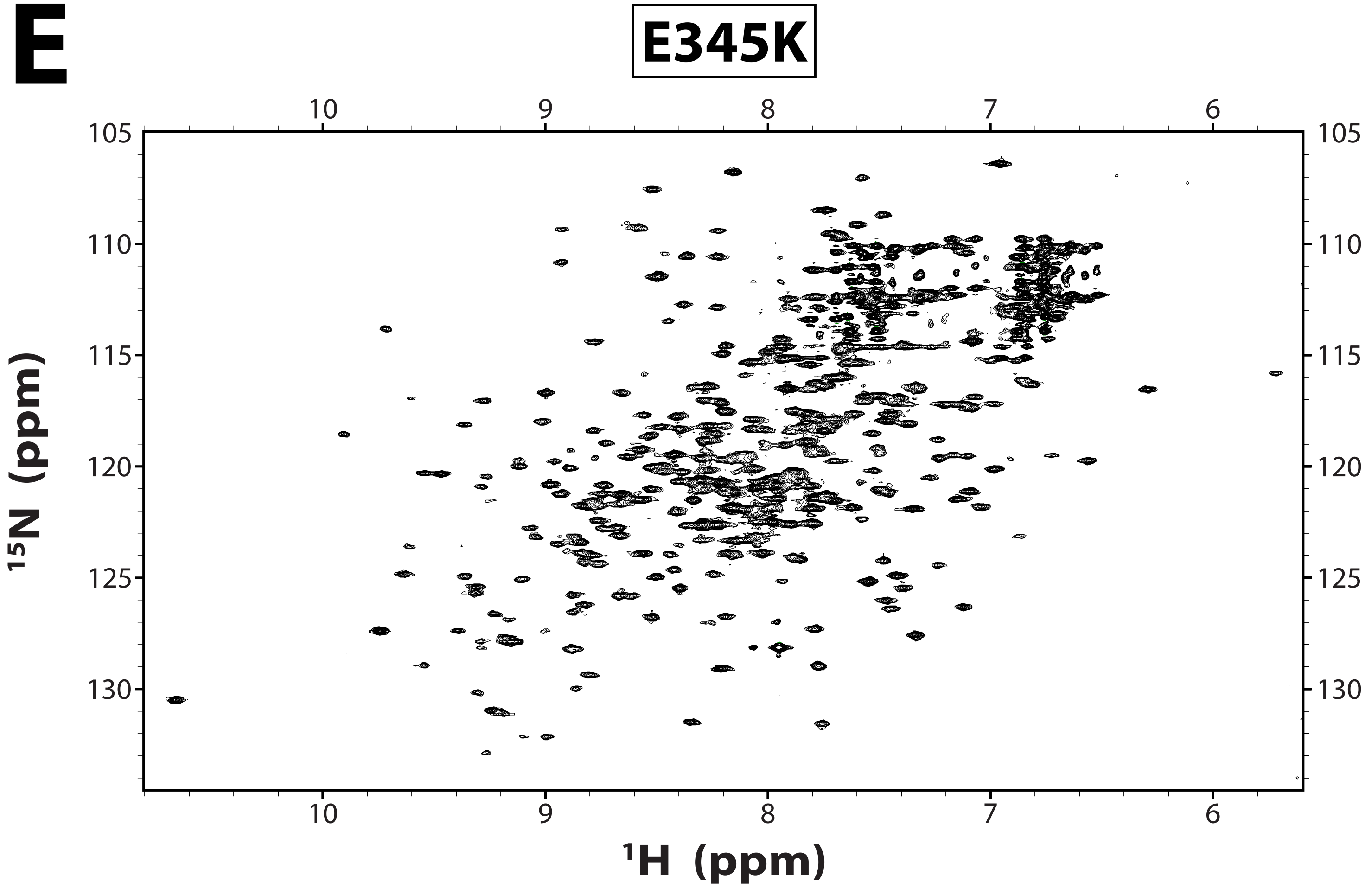

Figure S1

F

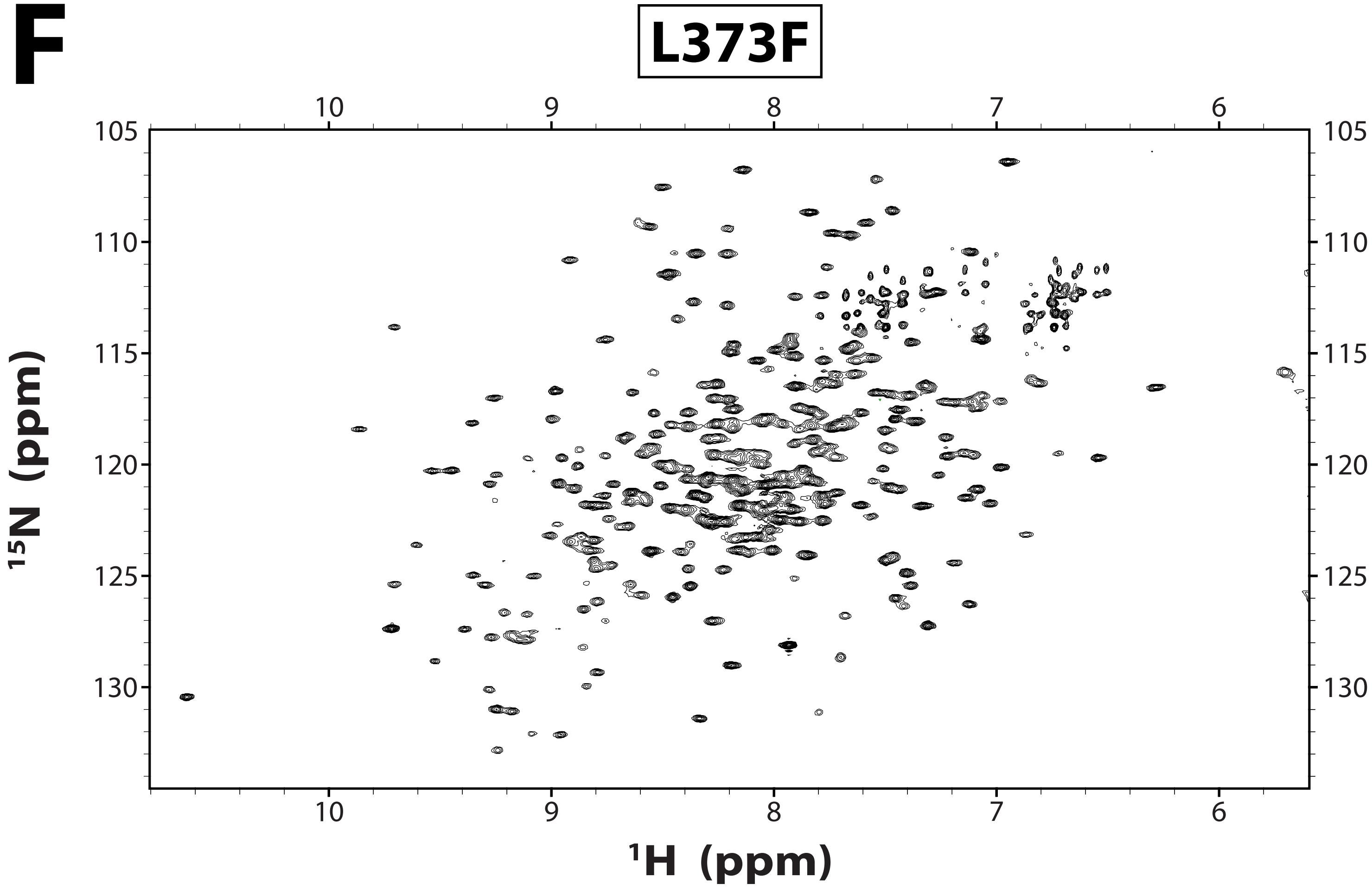

Figure S1

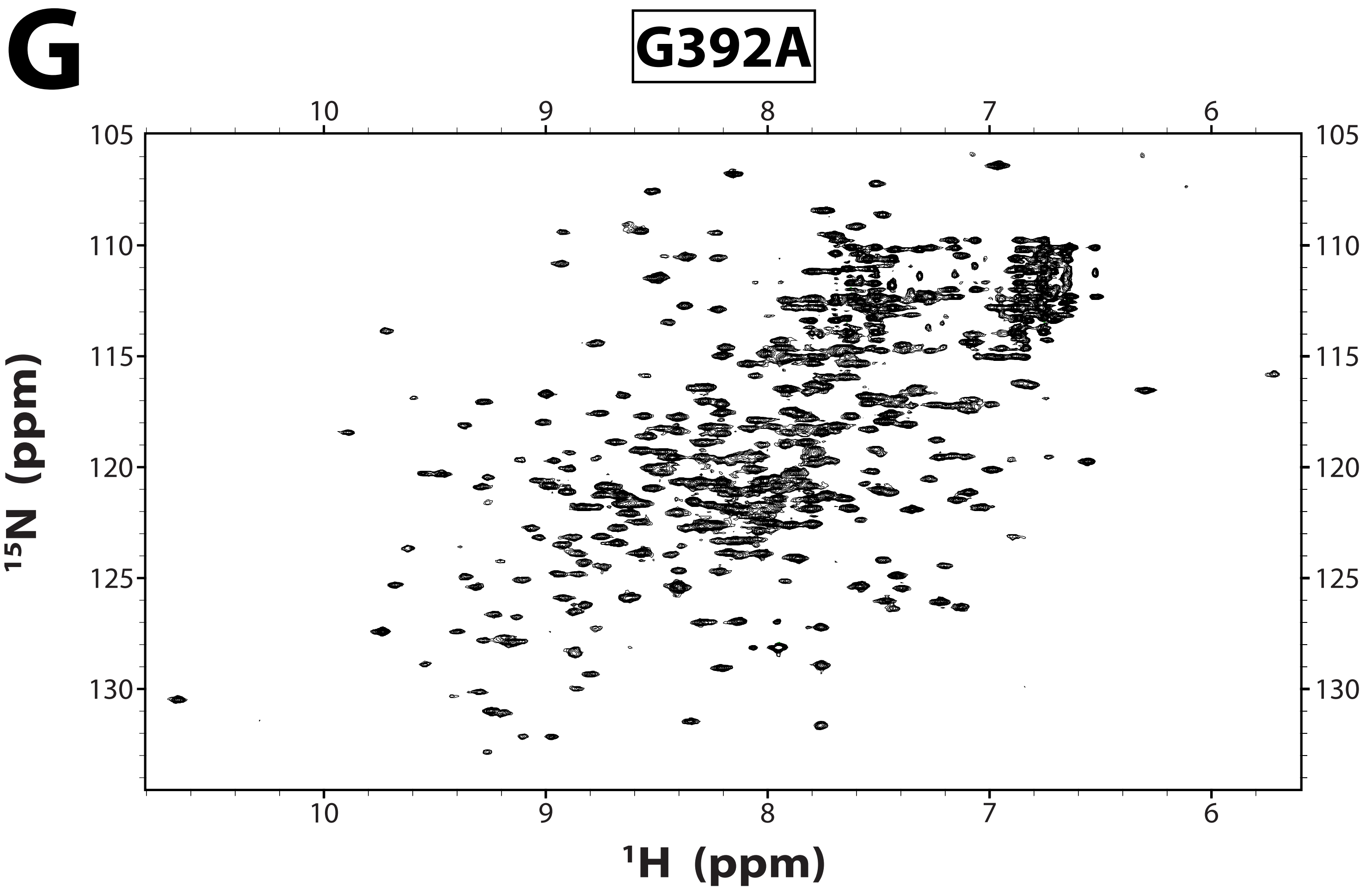

Figure S1

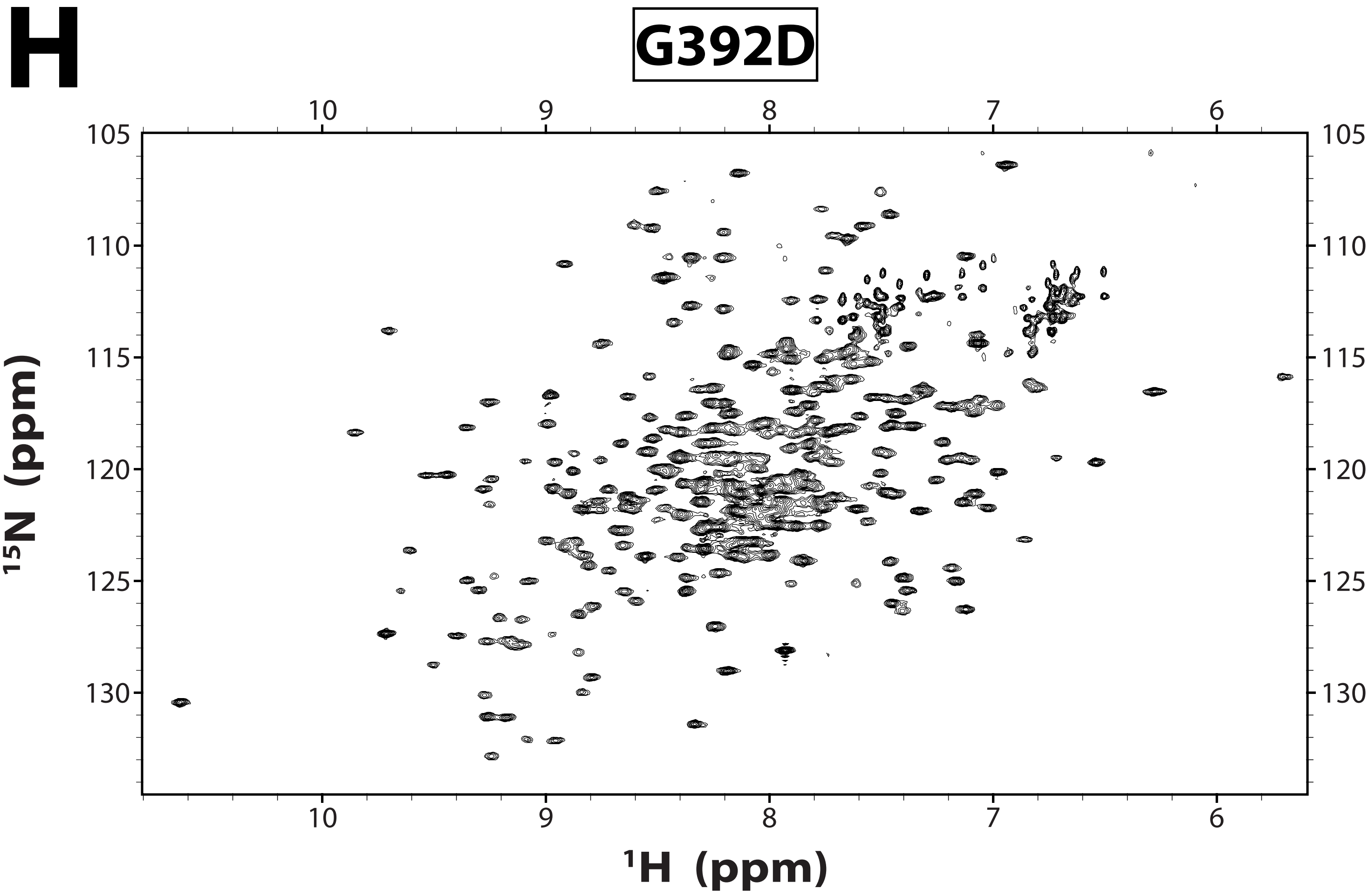

Figure S1

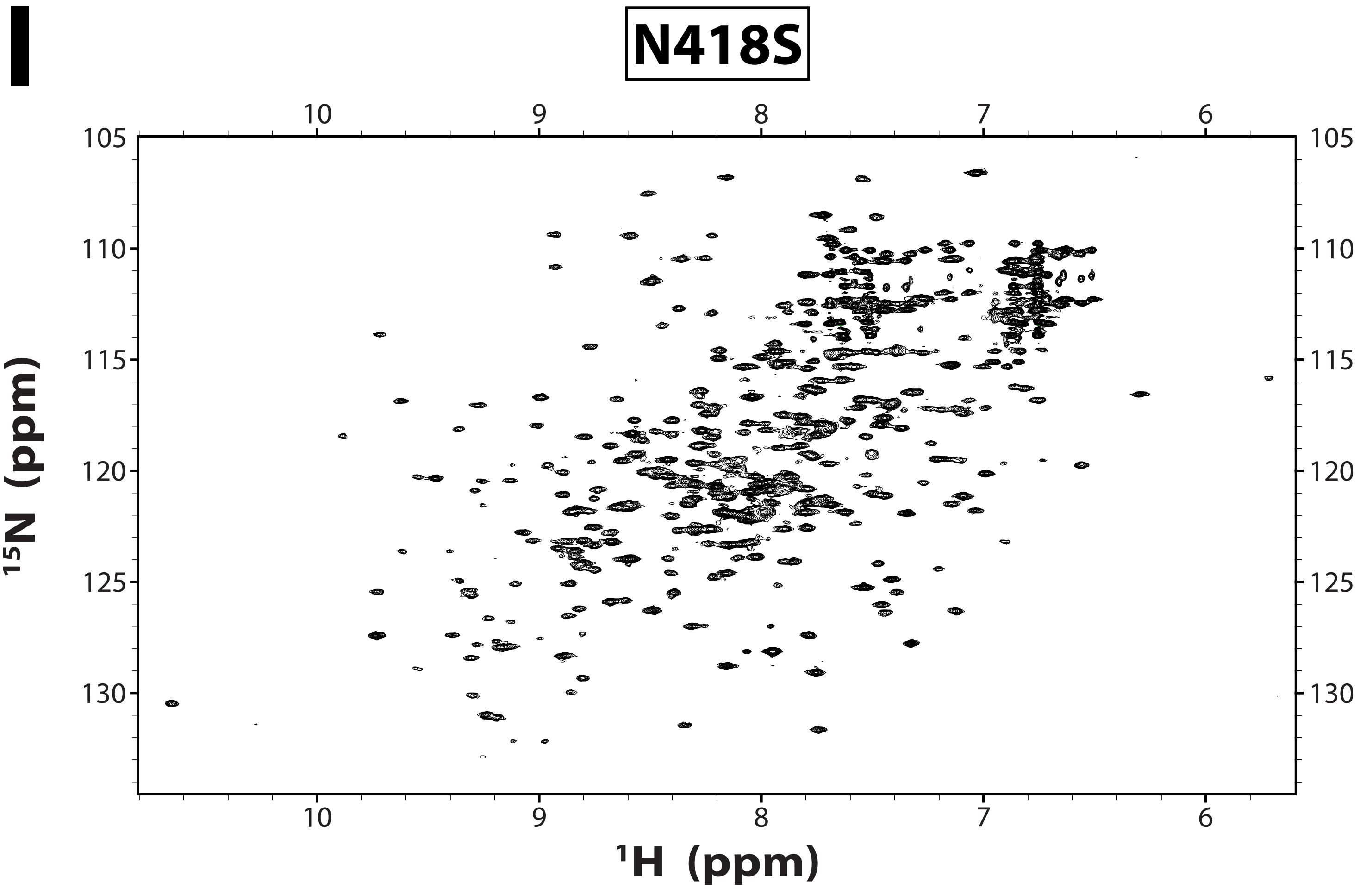

Figure S1

J

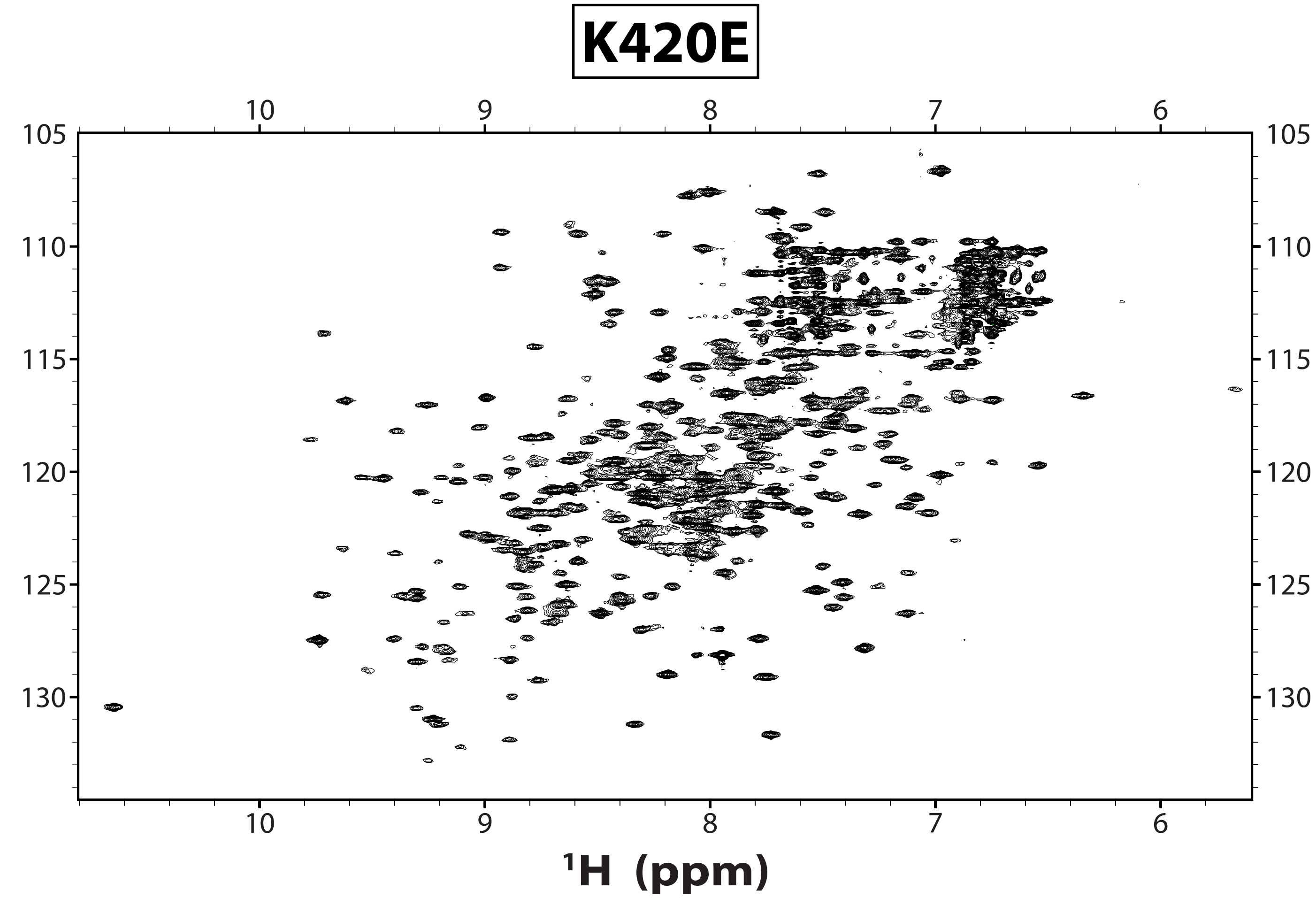

Figure S1

K

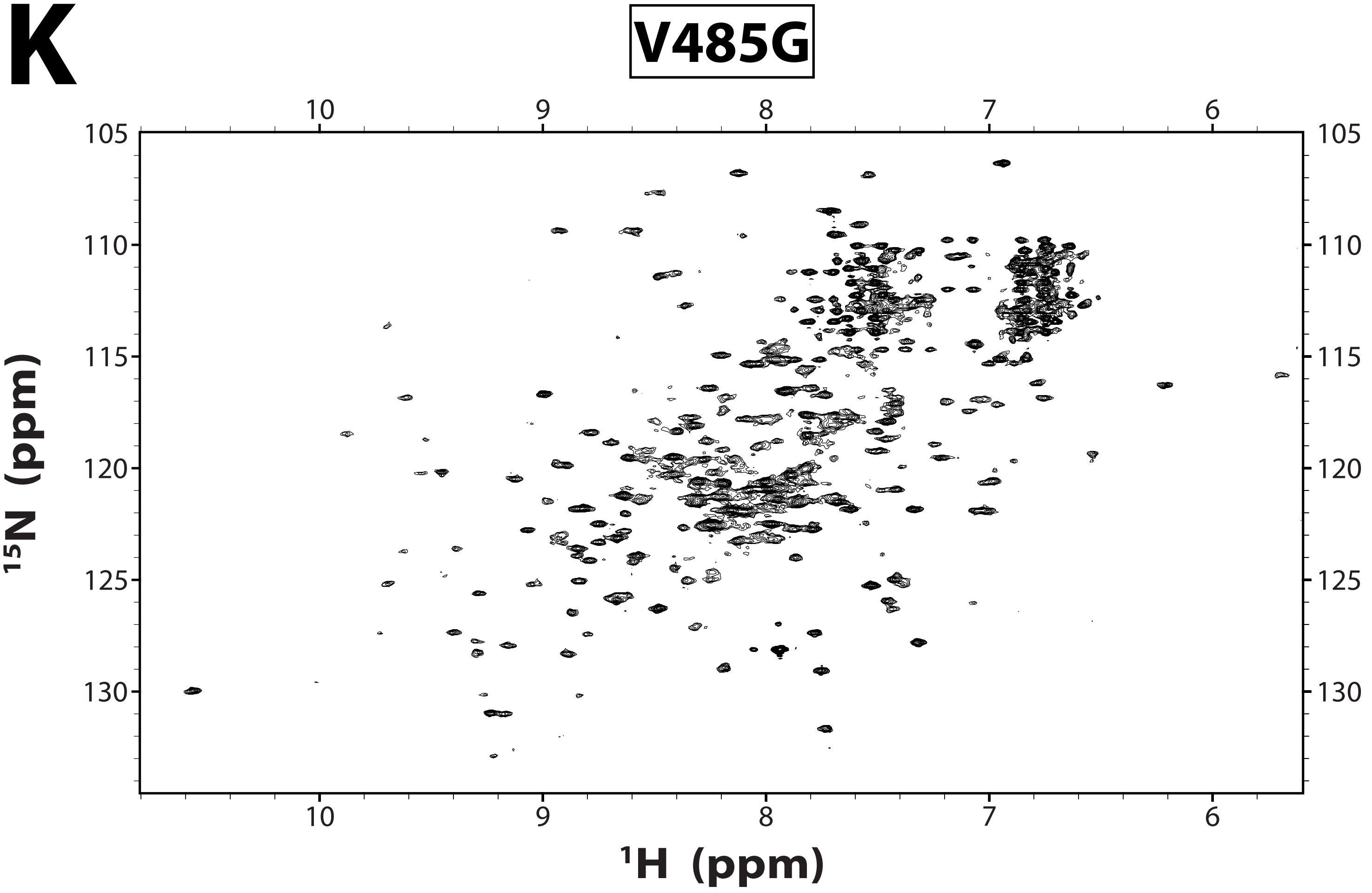

Figure S1. NMR spectra of USP7 variants associated with Hao-Fountain syndrome.
2D ^{15}N TROSY spectra of ^{15}N-labeled USP7 catalytic domain and its Hao-Fountain syndrome variants. Individual spectra are labeled with the corresponding variant name.

Figure S2

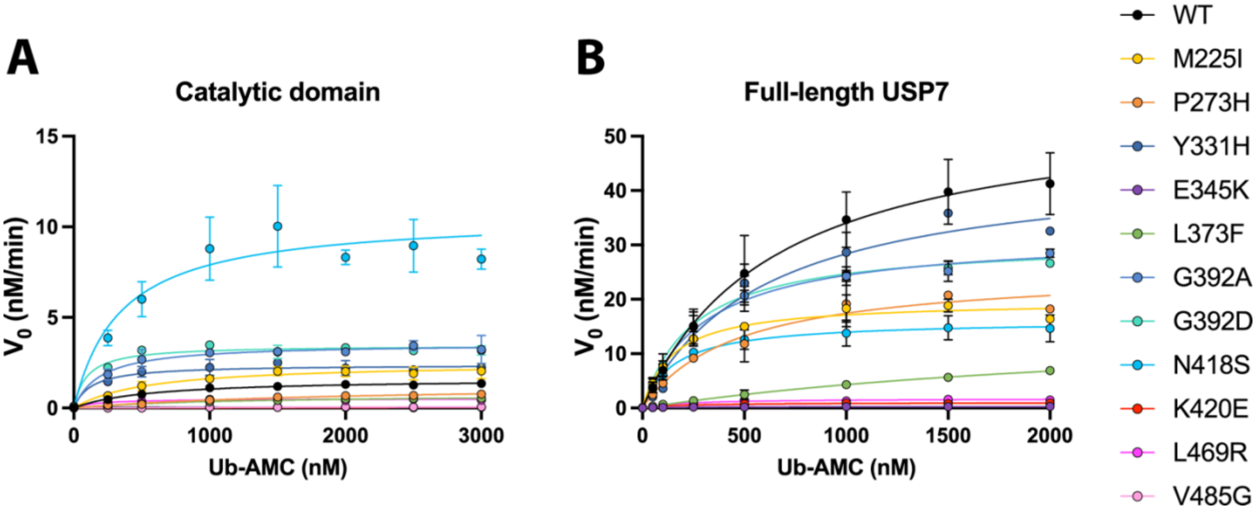

Figure S2. Effect of Hao-Fountain syndrome variants on USP7 activity.

Michaelis-Menten plots of the initial velocity (V_0) as a function of ubiquitin-AMC concentration shown for (**A**) USP7 catalytic domain and its mutants and (**B)** FL-USP7 and its mutants.

Figure S3

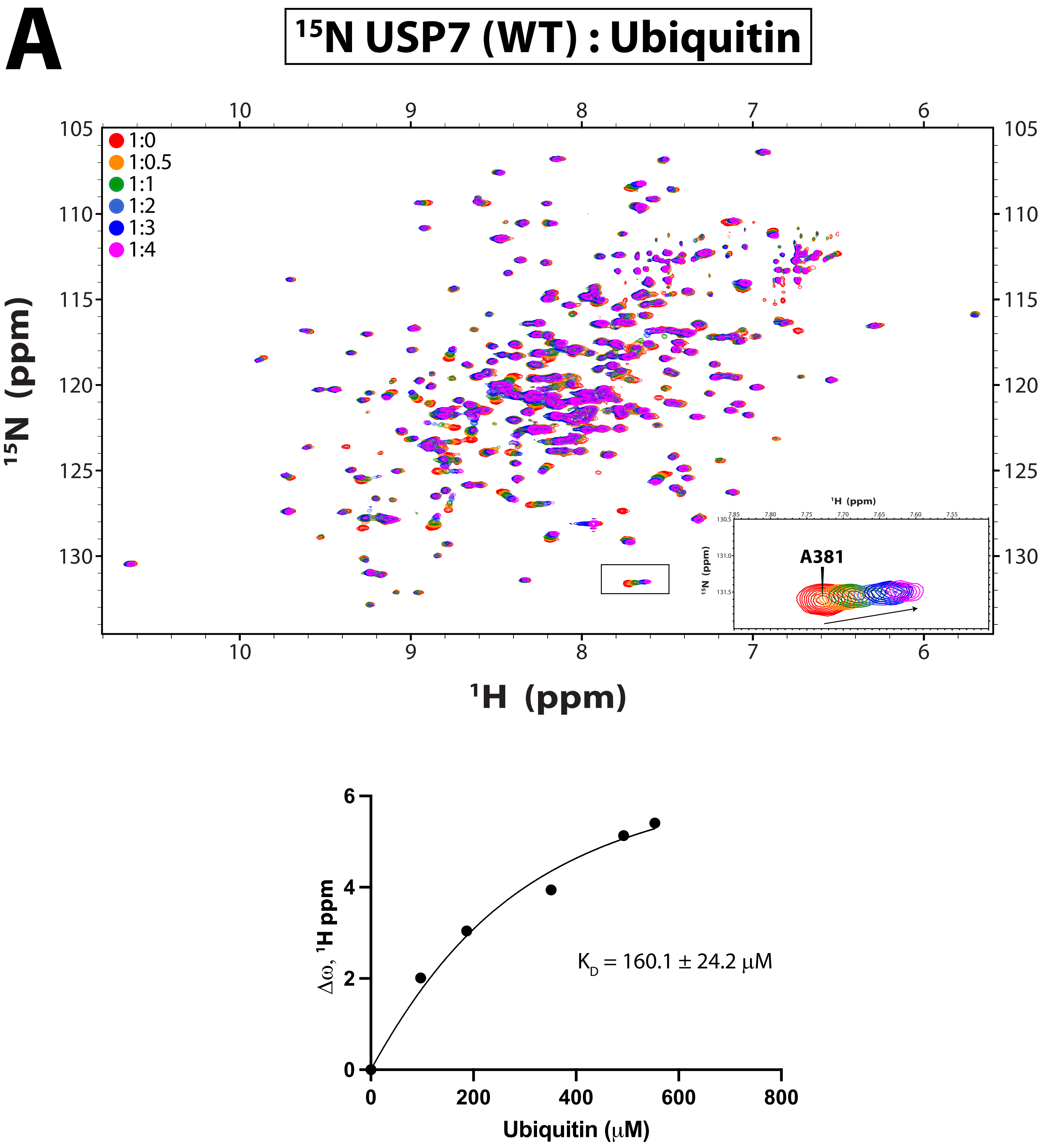
A
15N USP7 (WT) : Ubiquitin
1:0
1:0.5
1:1
1:2
1:3
1:4
1H (ppm)
15N (ppm)
A381
Δω, 1H ppm
Ubiquitin (μM)
K_D = 160.1 ± 24.2 μM

Figure S3

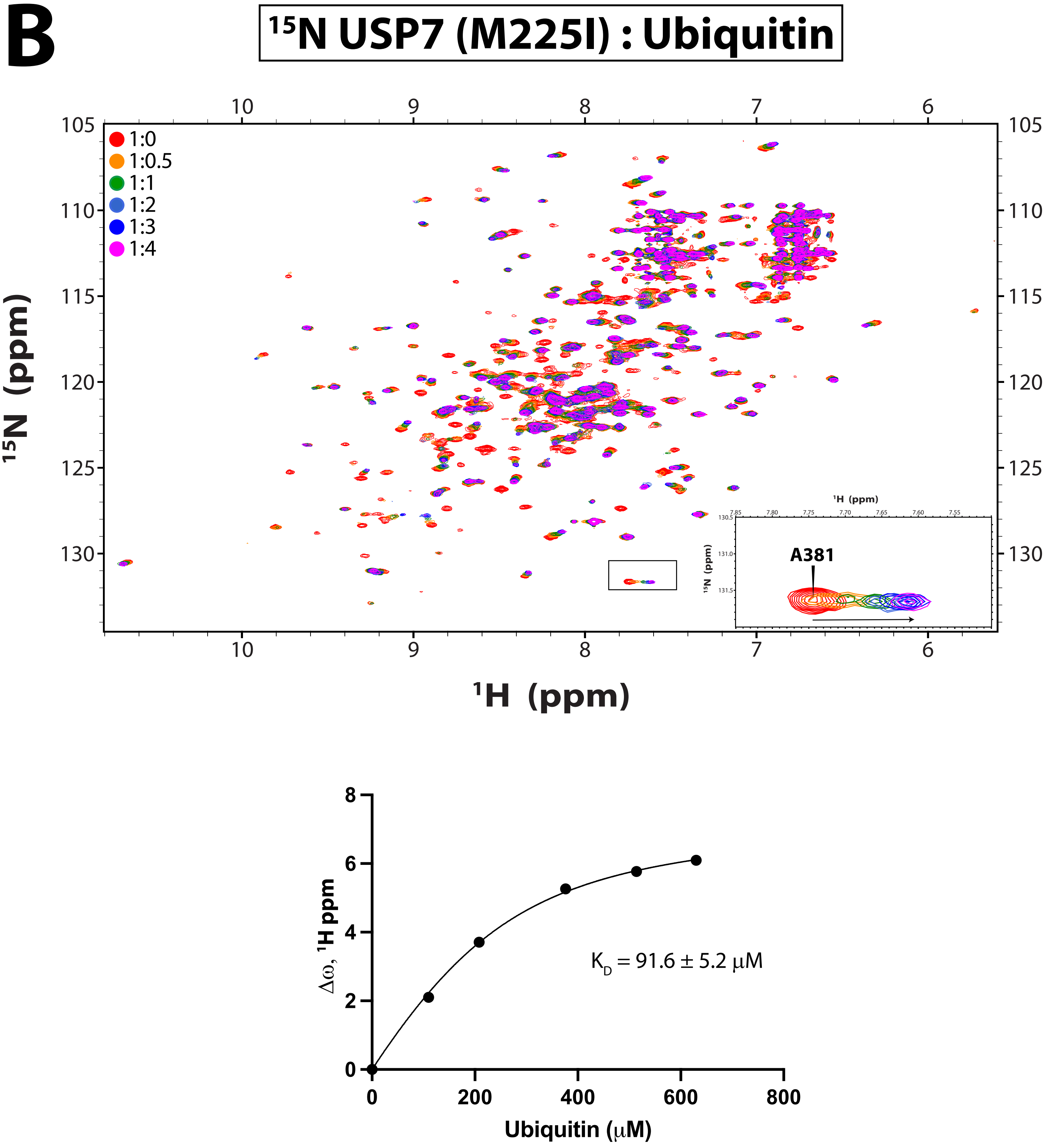
B
15N USP7 (M225I) : Ubiquitin
1:0
1:0.5
1:1
1:2
1:3
1:4
15N (ppm)
1H (ppm)
A381
K_D = 91.6 ± 5.2 μM
Δω, 1H ppm
Ubiquitin (μM)

Figure S3

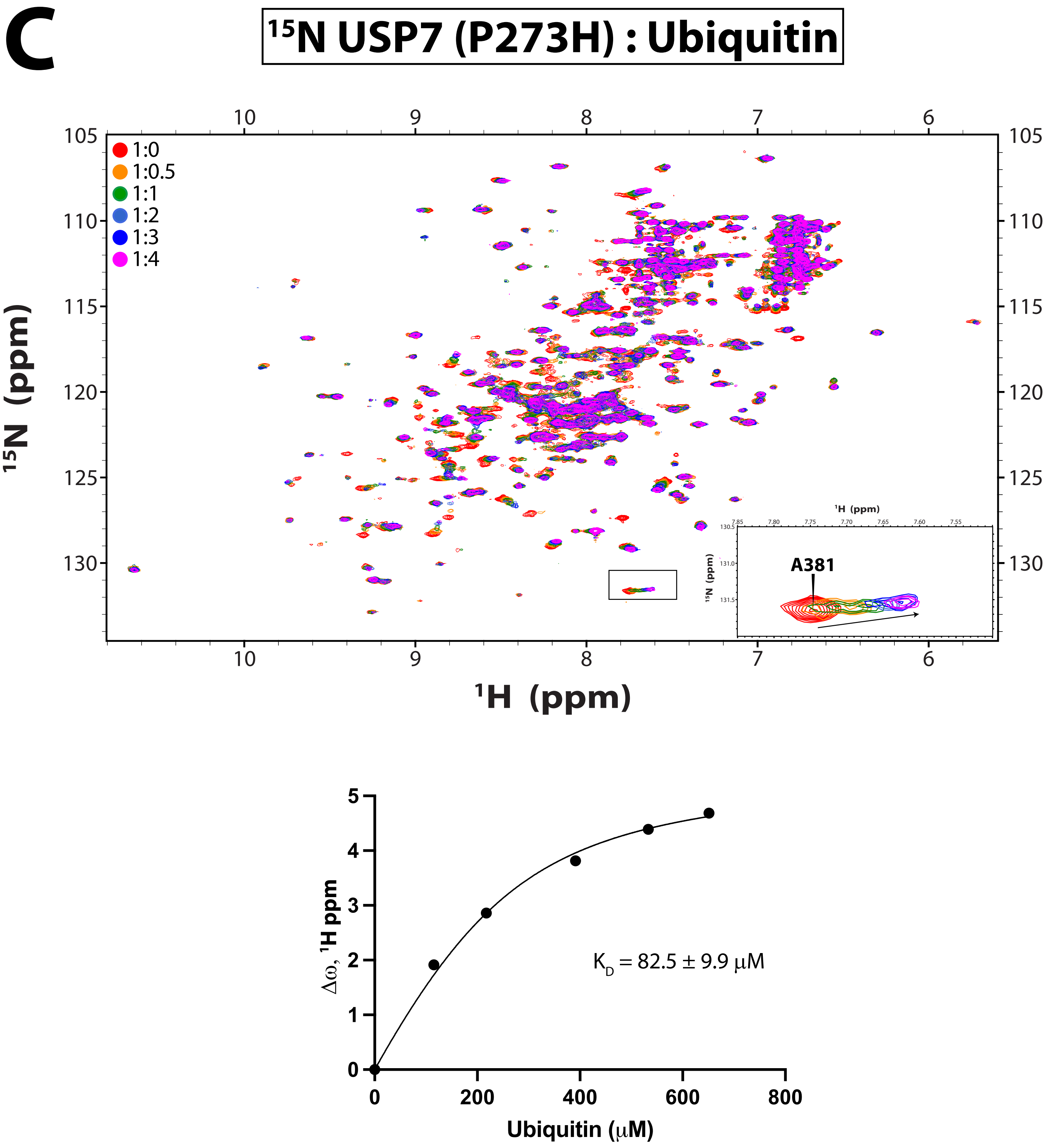

C
15N USP7 (P273H) : Ubiquitin
1:0
1:0.5
1:1
1:2
1:3
1:4
A381
1H (ppm)
15N (ppm)
Δω, 1H ppm
K_D = 82.5 ± 9.9 μM
Ubiquitin (μM)

Figure S3

D

¹⁵N USP7 (Y331H) : Ubiquitin

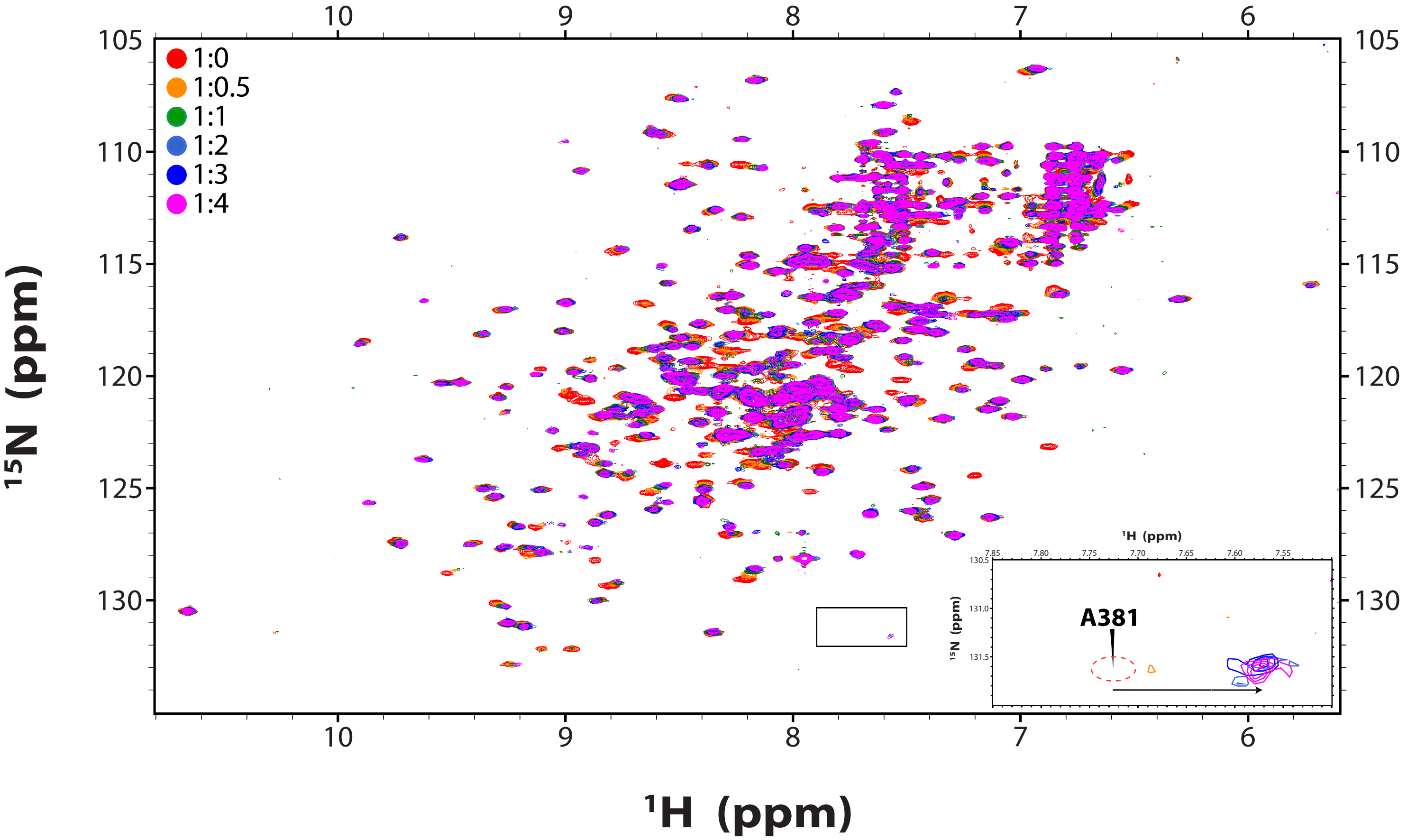

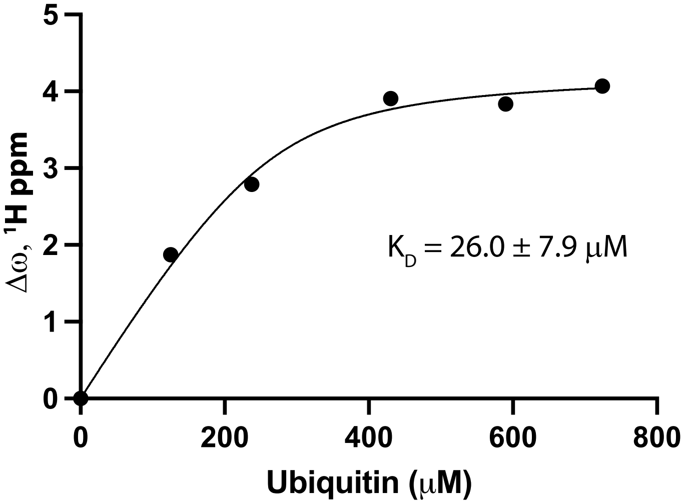

Figure S3

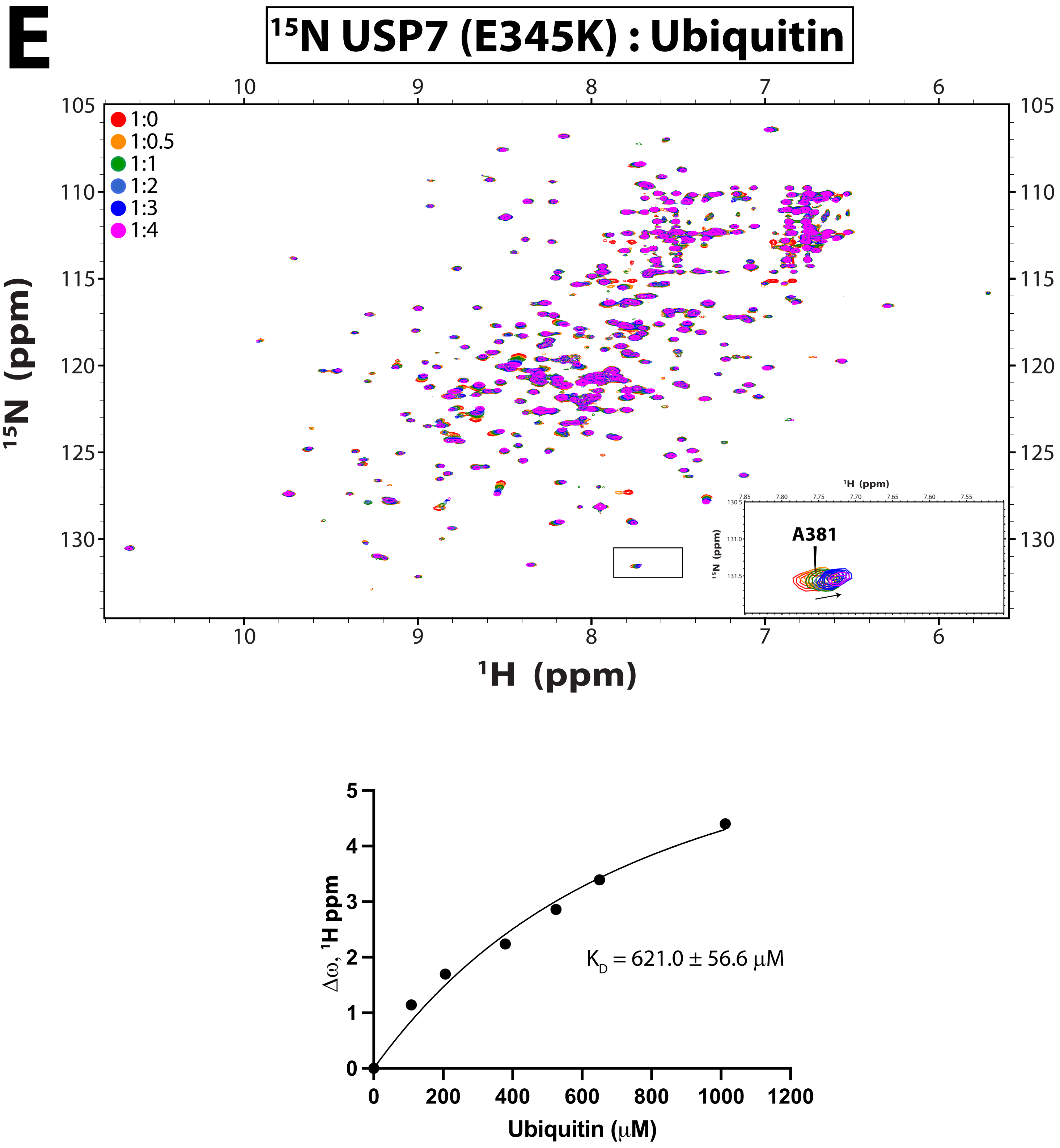
E
15N USP7 (E345K) : Ubiquitin
1:0
1:0.5
1:1
1:2
1:3
1:4
A381
15N (ppm)
1H (ppm)
Δω, 1H ppm
Ubiquitin (μM)
KD = 621.0 ± 56.6 μM

Figure S3

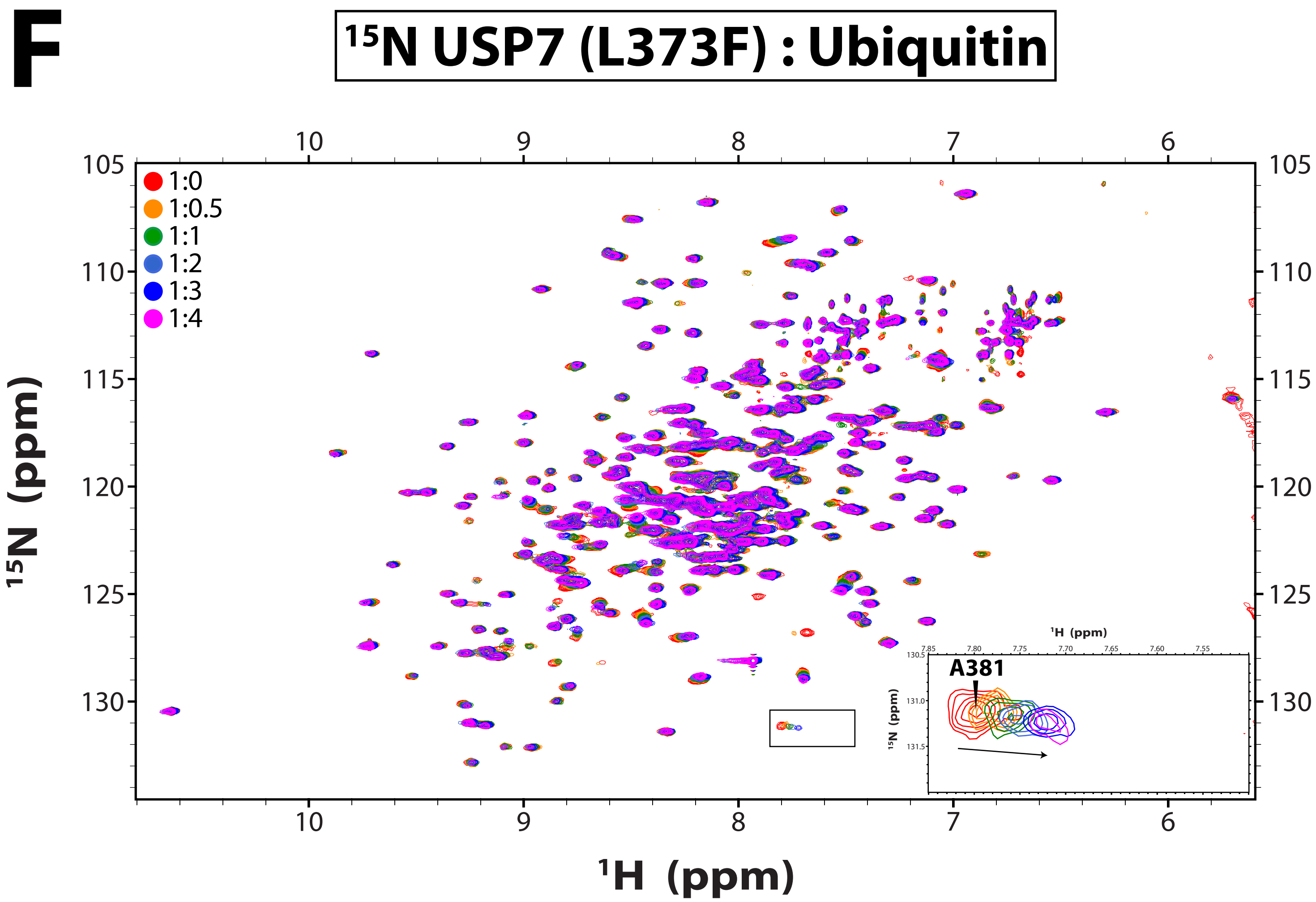

F
15N USP7 (L373F) : Ubiquitin
1:0
1:0.5
1:1
1:2
1:3
1:4
A381
15N (ppm)
1H (ppm)

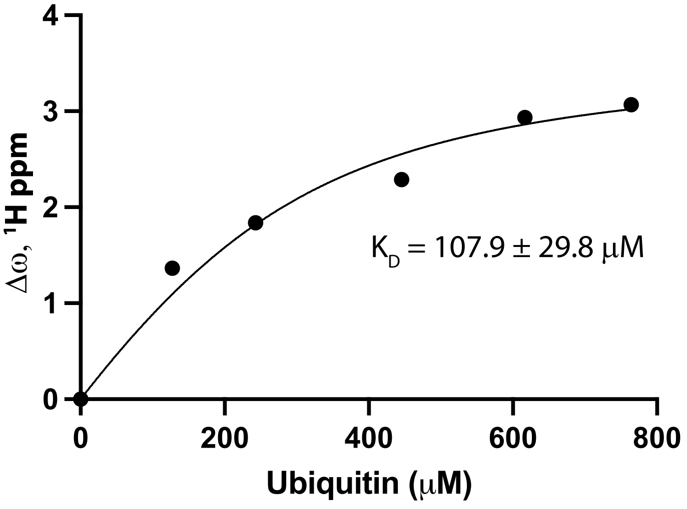

K_D = 107.9 ± 29.8 μM
Δω, 1H ppm
Ubiquitin (μM)

Figure S3

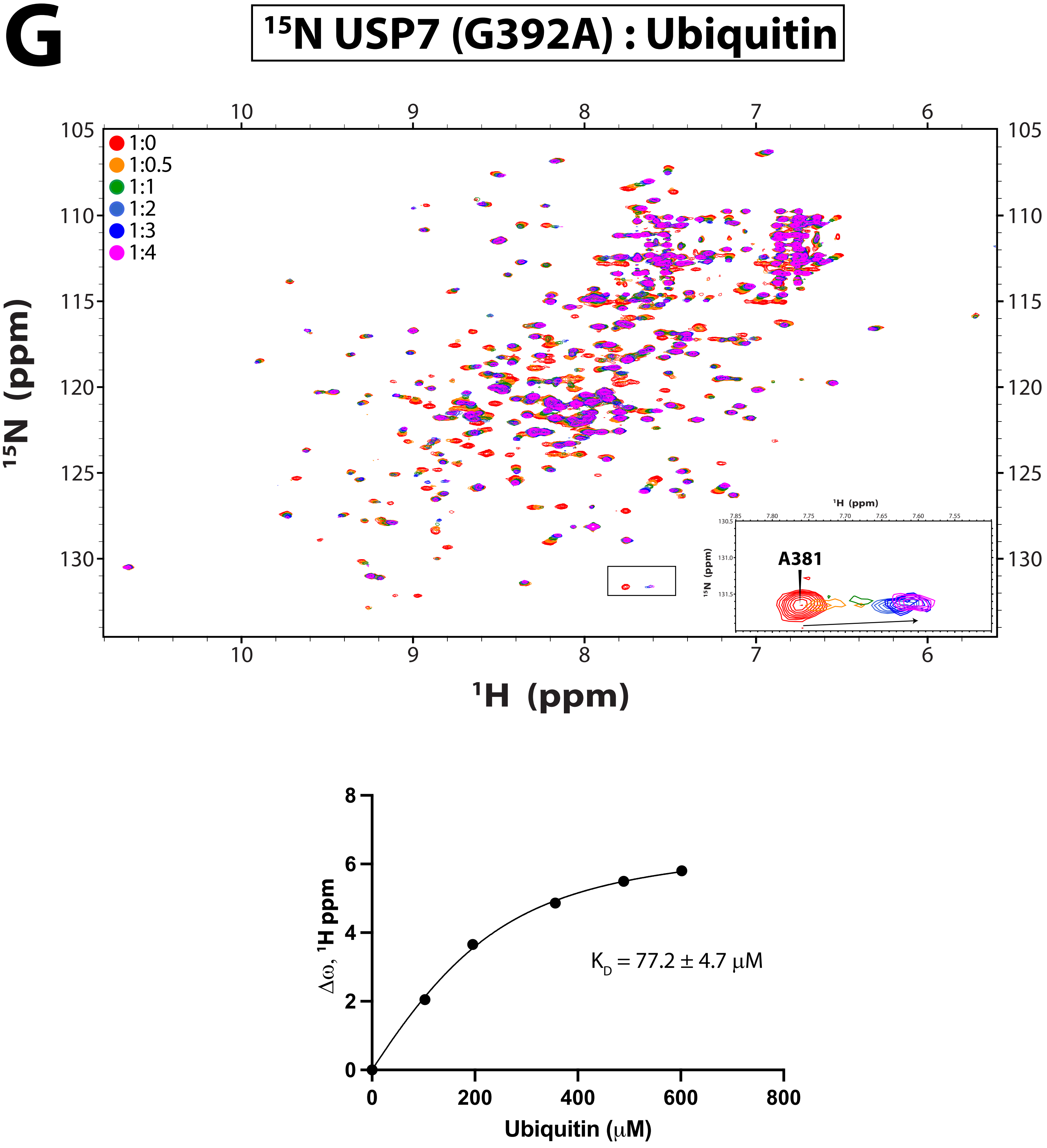
G
^{15}N USP7 (G392A) : Ubiquitin
1:0
1:0.5
1:1
1:2
1:3
1:4
^{15}N (ppm)
^{1}H (ppm)
A381
$\Delta\omega$, ^{1}H ppm
Ubiquitin (μM)
K_D = 77.2 ± 4.7 μM

Figure S3

H

^{15}N USP7 (G392D) : Ubiquitin

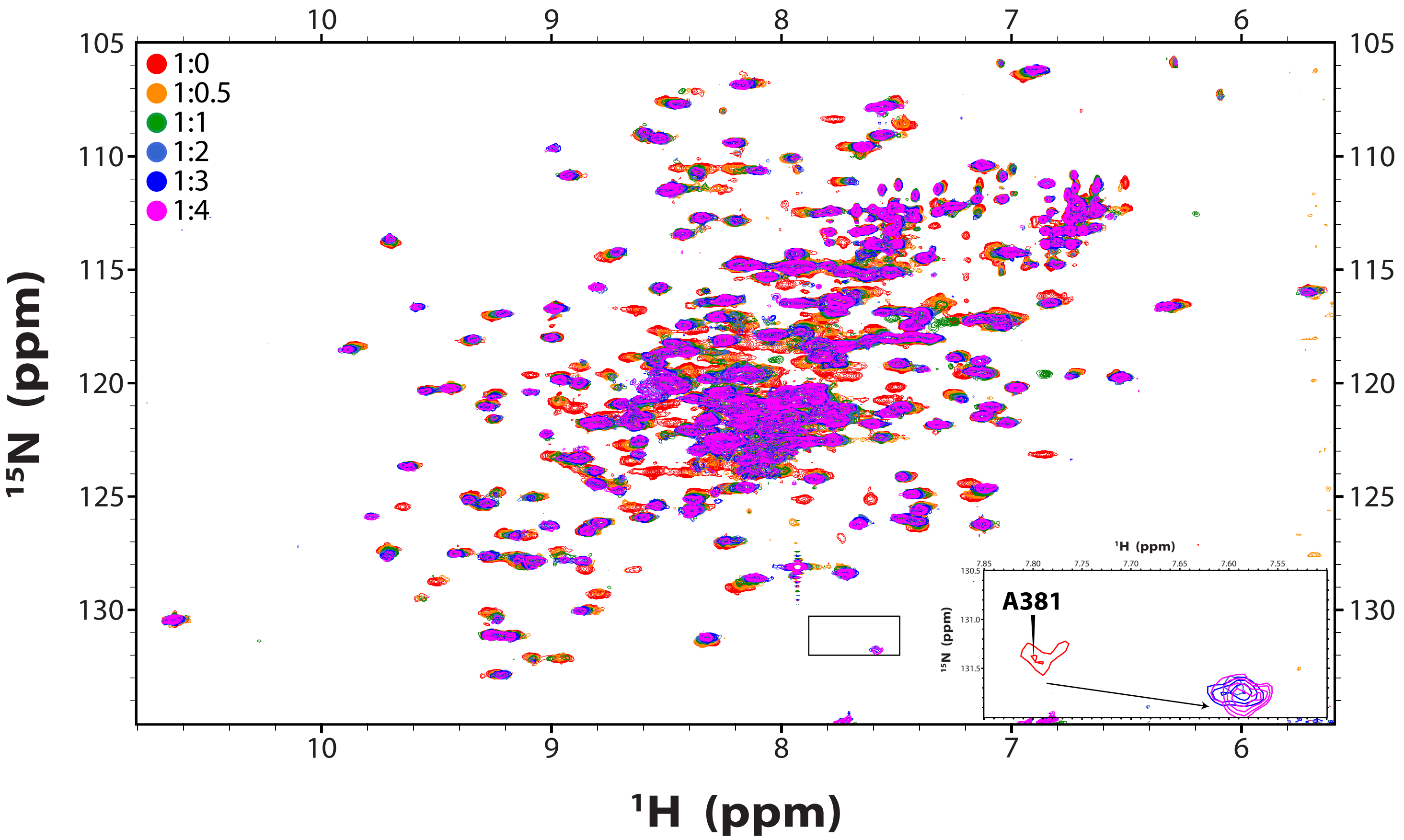

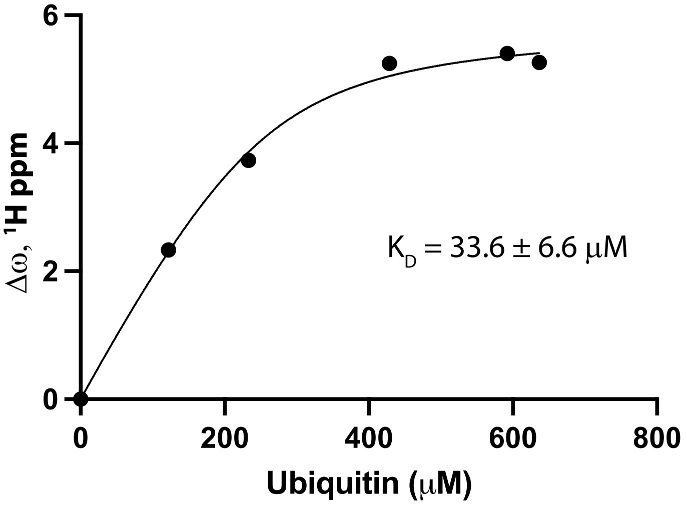

Figure S3

I

^{15}N USP7 (N418S) : Ubiquitin

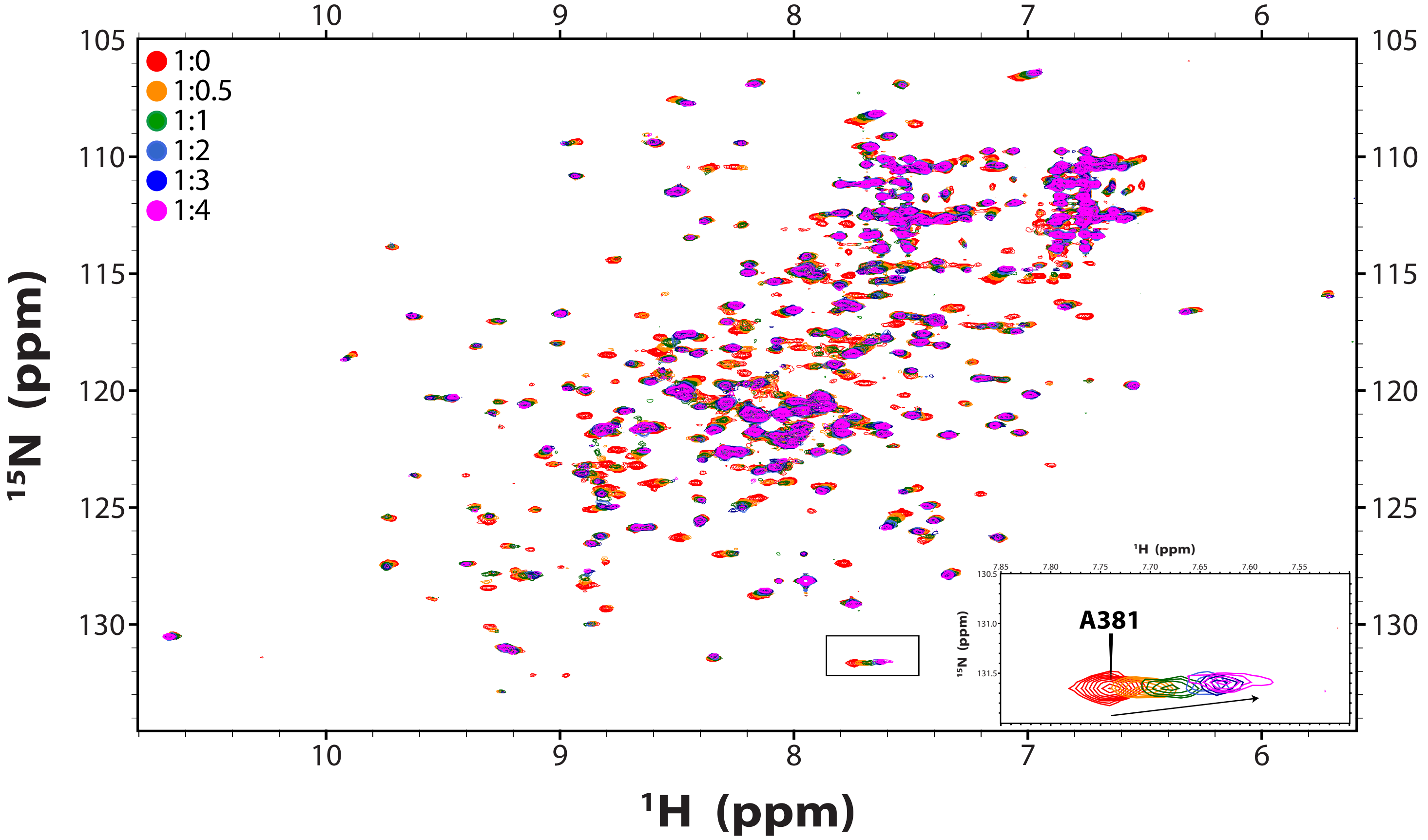

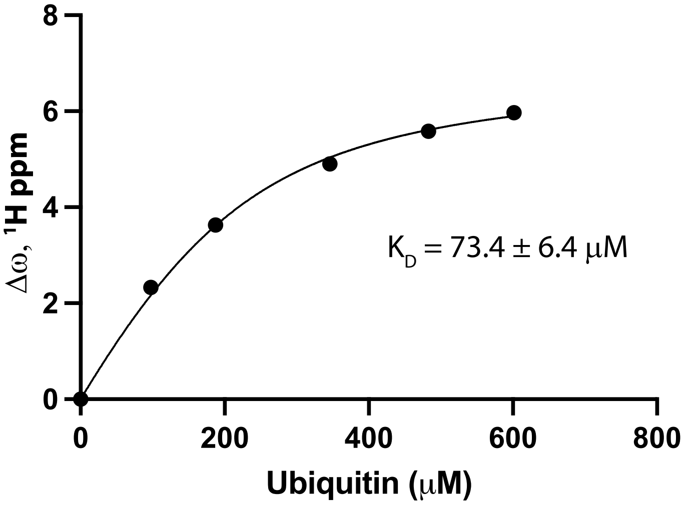

Figure S3

J

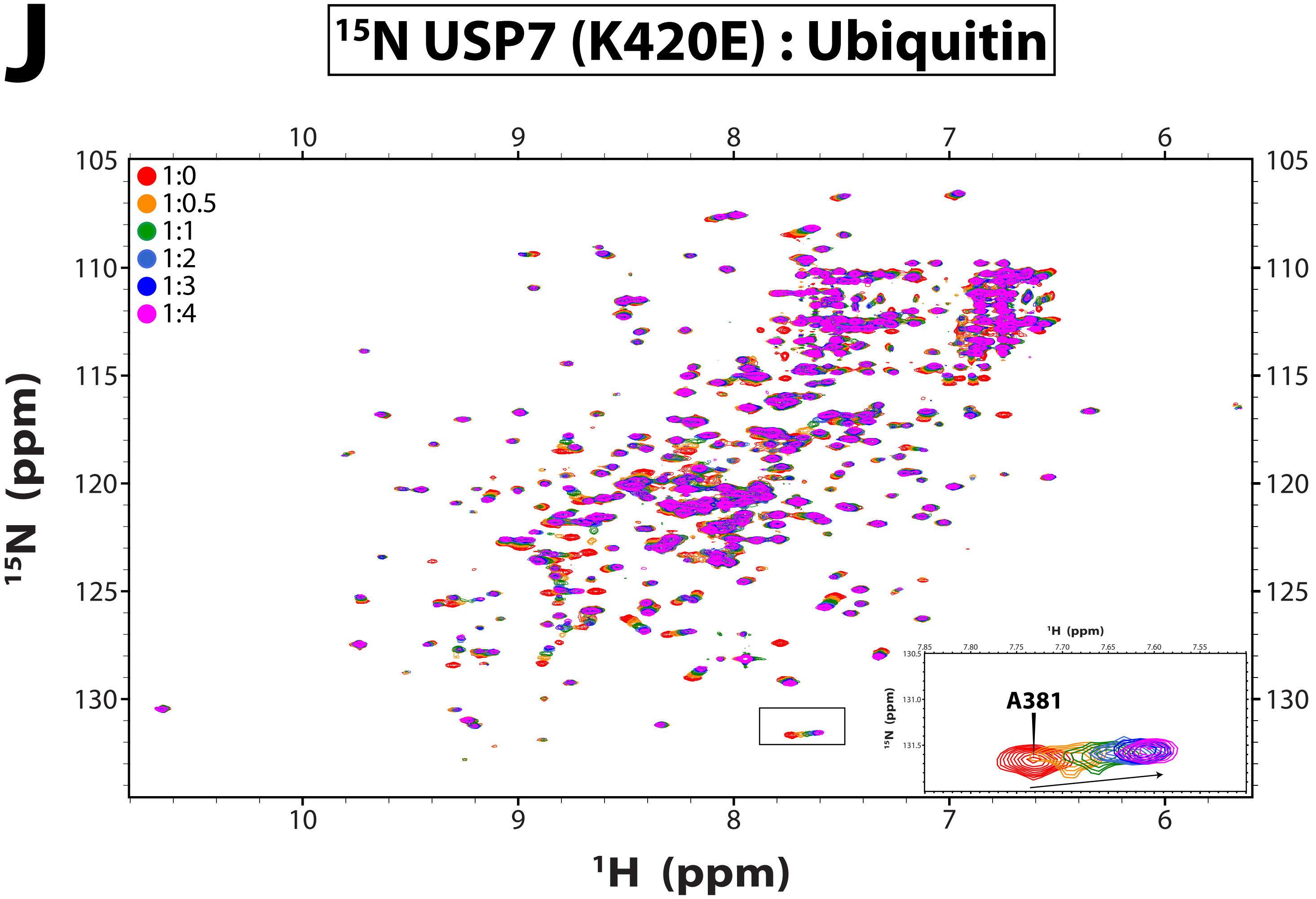

^{15}N USP7 (K420E) : Ubiquitin
1:0
1:0.5
1:1
1:2
1:3
1:4
A381
^{1}H (ppm)
^{15}N (ppm)

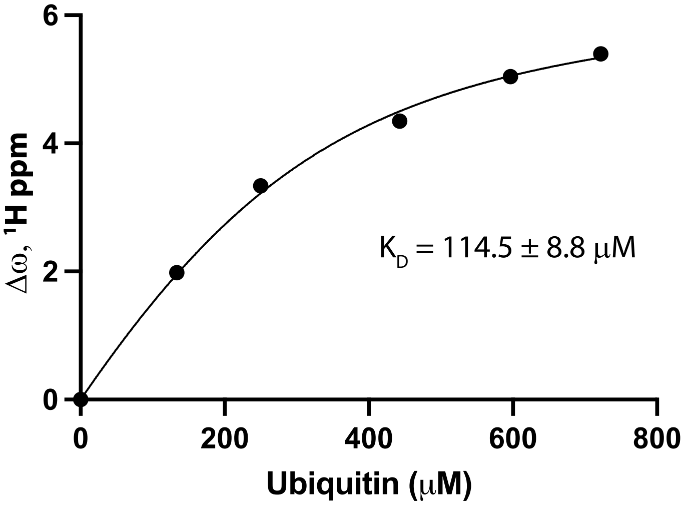

K_D = 114.5 ± 8.8 μM
Δω, ^{1}H ppm
Ubiquitin (μM)

Figure S3

K

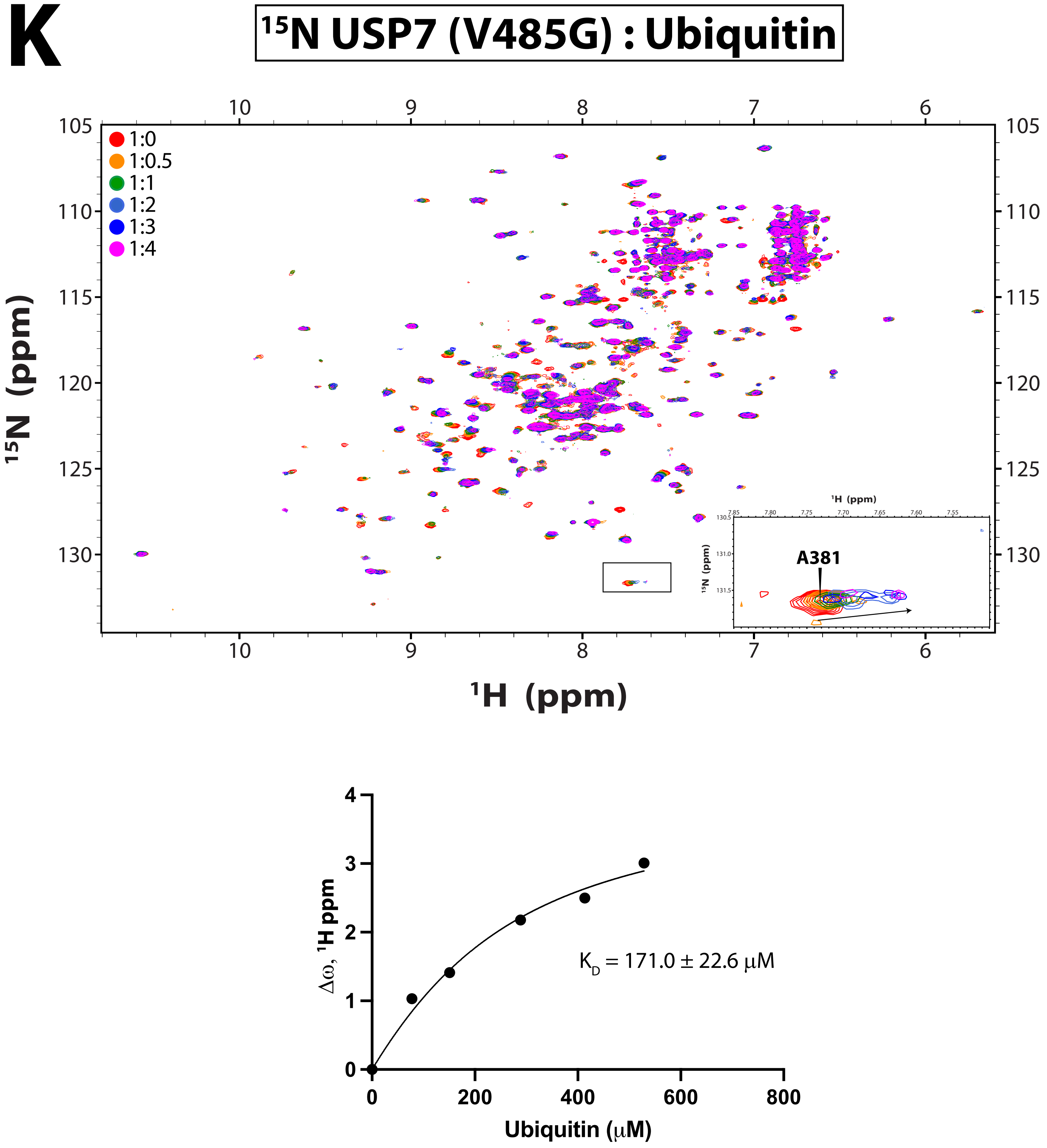

15N USP7 (V485G) : Ubiquitin
1:0
1:0.5
1:1
1:2
1:3
1:4
A381
15N (ppm)
1H (ppm)
KD = 171.0 ± 22.6 μM
Δω, 1H ppm
Ubiquitin (μM)

Figure S3. Ubiquitin binding to catalytic domains of USP7.

Top: ^{15}N TROSY spectra of the ^{15}N-labeled USP7 catalytic domain and its mutants gradually titrated with unlabeled ubiquitin. Residue A381 is showcased for each spectrum. USP7:ubiquitin molar ratios are shown. **Bottom:** Plot showing the global chemical shift perturbations ($\Delta\omega$) in the spectra as a function of ubiquitin concentration, used to estimate the binding affinities for each USP7 variant (K_D).

Figure S4

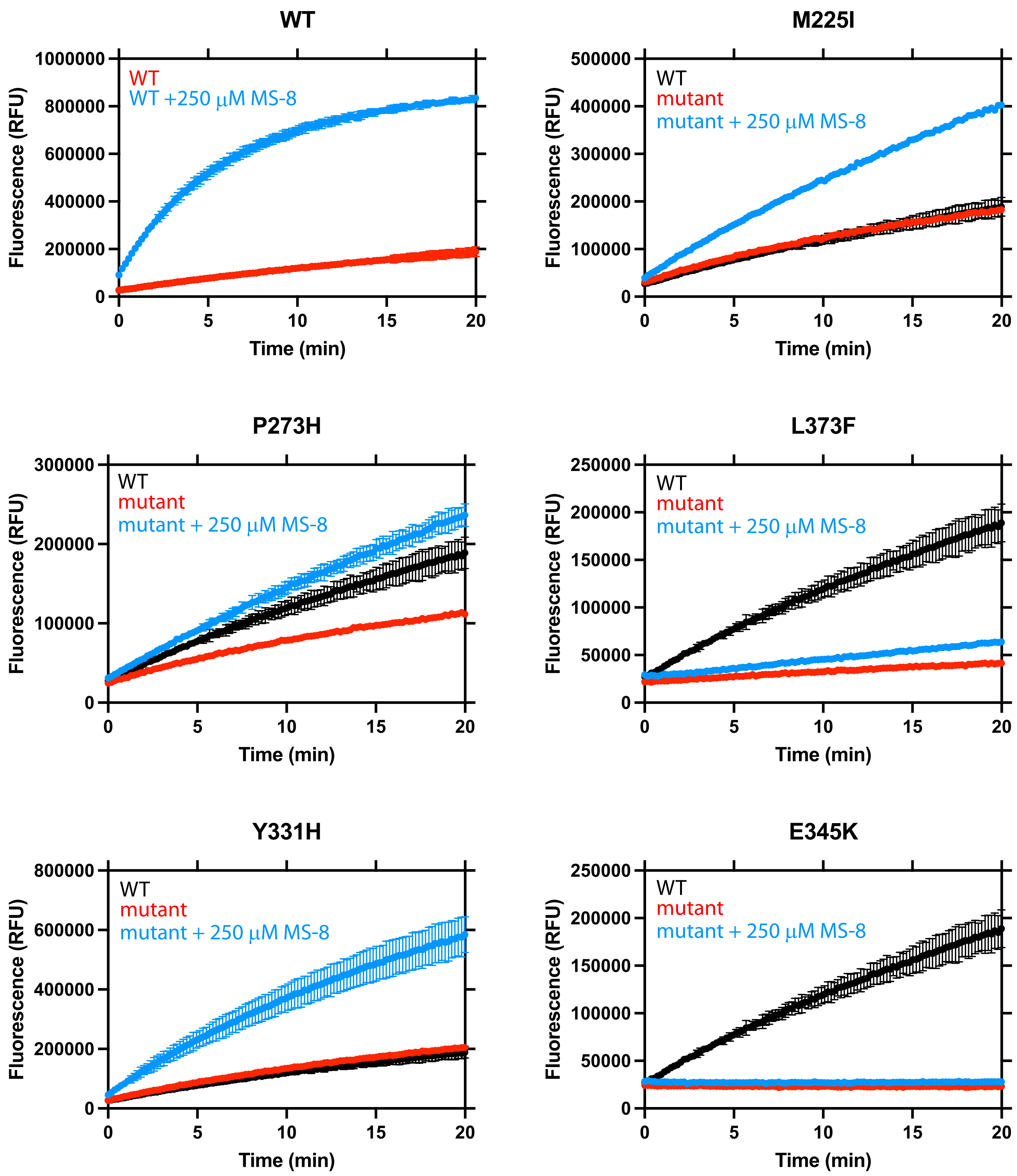

WT
WT
WT +250 μM MS-8
Fluorescence (RFU)
1000000
800000
600000
400000
200000
0
0
5
10
15
20
Time (min)
M225I
WT
mutant
mutant + 250 μM MS-8
500000
400000
300000
200000
100000
P273H
300000
200000
100000
L373F
250000
200000
150000
100000
50000
Y331H
800000
600000
400000
200000
E345K
250000
200000
150000
100000
50000

Figure S4 (cont.)

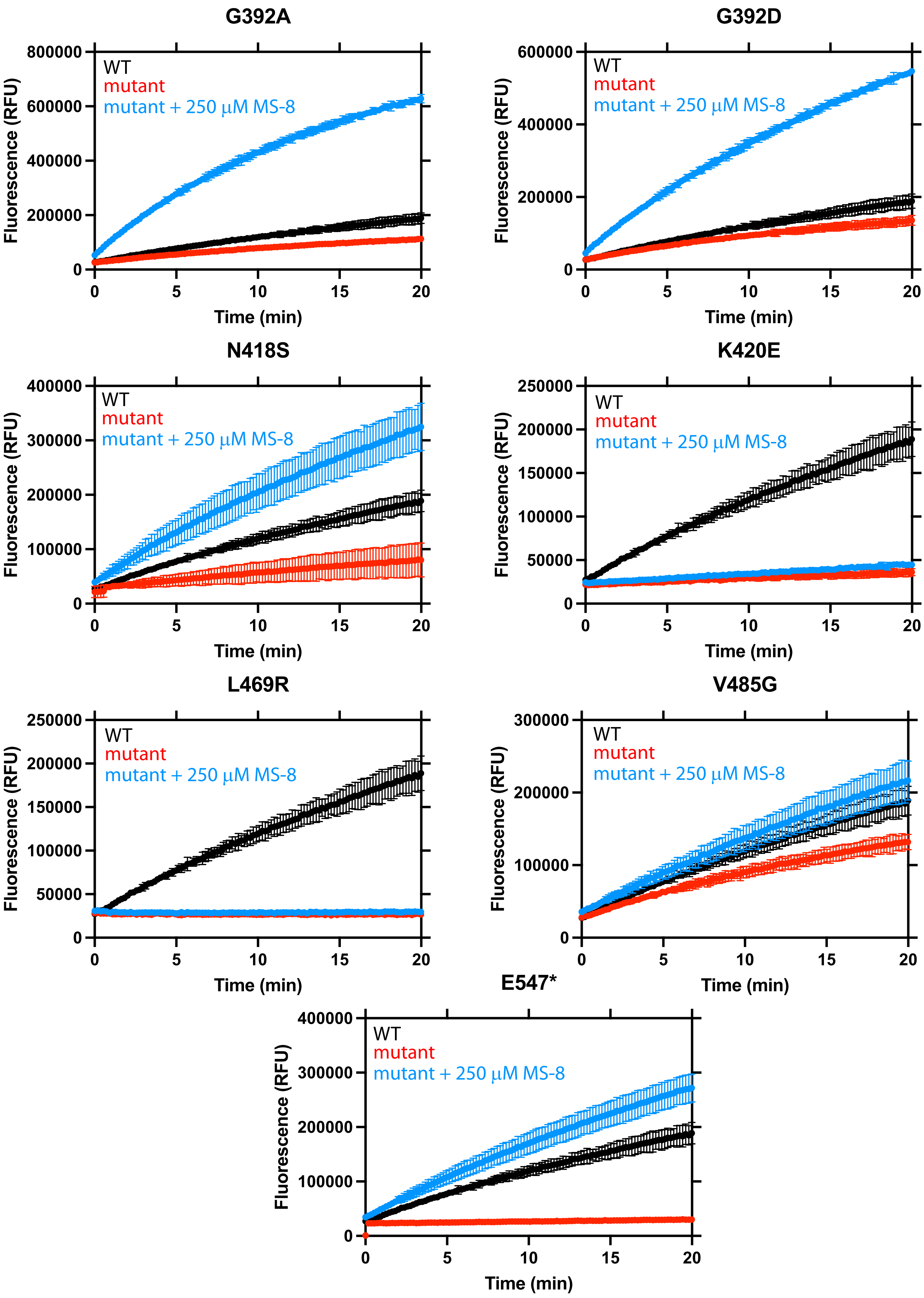

Figure S4. MS-8 enhances the activity of USP7 variants.
Comparison of the time course of deubiquitination reaction for FL-USP7 variants alone (red) and treated with 250 μM MS-8 (blue). The untreated WT curve is shown for reference (black). 0.1 nM USP7 was used with 500 nM ubiquitin-rhodamine as its fluorogenic substrate.

Table S1

Summary of enzyme kinetics and ubiquitin-binding affinities of USP7 mutations associated with Hao-Fountain syndrome.

	WT	M225I	P273H	Y331H	E345K	L373F	G392A	G392D	N418S	K420E	V485G	L469R
**Catalytic domain**												
k_{cat} (min^{-1})	1.6±0.0	2.5±0.2	1.3± 0.2	2.4±0.1	0.1±0.0	0.8±0.0	3.6±0.2	3.5±0.1	10.6±0.7	N/A	0.2±0.0	0.5±0.0
K_M (μM)	0.6± 0.1	0.6±0.1	1.9±0.4	0.1±0.1	N/A	1.1± 0.2	0.2±0.1	0.1±0.0	0.3±0.1	N/A	N/A	0.2± 0.1
k_{cat}/K_M ($min^{-1}μM^{-1}$)	2.9±0.3	4.6±1.1	0.7±0.2	18.6±7.0	N/A	0.7±0.1	17.8±4.8	35.9±11.4	30.9±9.2	N/A	N/A	2.9±1.1
K_D (μM)	160.1±24.2	91.6±5.2	82.5±9.9	26.0±7.9	621.0±56.6	107.9±29.8	77.2±4.7	33.6±6.6	73.4±6.4	114.5±8.8	171.0±22.6	N/A
**Full-length**												
k_{cat} (min^{-1})	56.2±3.4	25.5± 2.7	19.7±0.7	45.3±3.4	0.2± 0.0	16.1±3.1	31.9±1.1	30.9±1.5	16.1±0.7	1.0±0.1	1.3±0.1	1.8±0.2
K_M (μM)	0.7±0.1	0.5±0.1	0.2±0.0	0.6±0.1	N/A	2.8±0.8	0.3±0.0	0.3±0.0	0.2±0.0	N/A	N/A	0.21±0.1
k_{cat}/K_M ($min^{-1}μM^{-1}$)	86.3±14.4	55.1±16.4	132.0±20.8	76.0±15.9	N/A	5.9±2.0	104±13.1	123.1±21.6	102.5±20.2	N/A	N/A	8.2±3.0
